# Pediatric Dental Trauma:Wide Horizon of Ignored Etiological Factors

**DOI:** 10.5005/jp-journals-10005-1090

**Published:** 2010-04-15

**Authors:** Rajesh T Anegundi, Shruthi B Patil, Shubha M, Raghavendra Havale

**Affiliations:** 1 Professor and Head, Department of Pediatric Dentistry, SDM College of Dental Sciences and Hospital, Dharwad, Karnataka, India; 2 Associate Professor, Department of Pediatric Dentistry, SDM College of Dental Sciences and Hospital, Dharwad, Karnataka, India; 3 Postgraduate Student, Department of Pediatric Dentistry, SDM College of Dental Sciences and Hospital, Dharwad, Karnataka, India; 4 Assistant Professor, Department of Pediatric Dentistry, AME’s Dental College, Raichur, Karnataka, India

**Keywords:** Dental trauma, Etiologies, Prevention.

## Abstract

Trauma of the oral and peroral structures are one of the most common and frequent complaints after dental caries with which a child is being referred to a dental clinic. As an emergency, we tend to treat the injuries without understanding or neglecting the cause of trauma. The different possible etiological factors are unnoticed, not revealed or not noted while taking the history of the patient. Sometimes negligence of the etiology by the dentist himself or the accompanying person could influence the prognosis and prevention. Thus, this paper is an effort towards exploring the common yet unnoticed etiological factors of pediatric dental trauma which we tend to knowingly ignore.

## INTRODUCTION

Dental trauma cases reported in childhood and adolescence are alarming.^1^ Orofacial injuries in children are a cause of important public dental health problems.^2^ In addition to the physiological outcome, economic and psychological components accompany these injuries.^3^ The etiologies for these injuries can be generalized as falls in infancy, child physical abuse, accidental falls and collisions, bicycle injuries, sports, automobile injuries, assaults, mental retardation, epilepsy, drug related injuries and dentinogenesis imperfecta.^4^ Among all these etiologies, accidental falls are often stated as the most common cause of dental injuries, but the event resulting in the occasional fall/collision is rarely reported or ignored.^5^ Similarly, there are some other common causes which are not given importance and neglected. A proportion of all these oral injuries can be prevented if the risk factors and etiologies are better understood. This knowledge is essential for developing and implementing effective prevention.^3^

The purpose of this article is to present the cases with unique etiologies reported to Department of Pediatric Dentistry, SDM Institute of Dental Sciences, Dharwad, Karnataka, India, and to refresh and assess the impact of different neglected/ignored etiologies on orofacial structures. The cases reported are summarized in Table 1.

## DISCUSSION

We are aware about the causes reported commonly, but the ignored ones. A convenient way to consider etiologic factors of trauma is to relate them to the chronological or developmental age. In preschool years (0-4 years) incidence of orofacial injuries results as the child learns to walk. Junior school years (5-11 years) falls resulting from play, running and slippage or colliding with another child are common. In secondary school years (11-18 years) sports related injuries are common which can be prevented by using effective measures.^1^ These etiologies are the commonly reported ones.

**Fig. 1:**
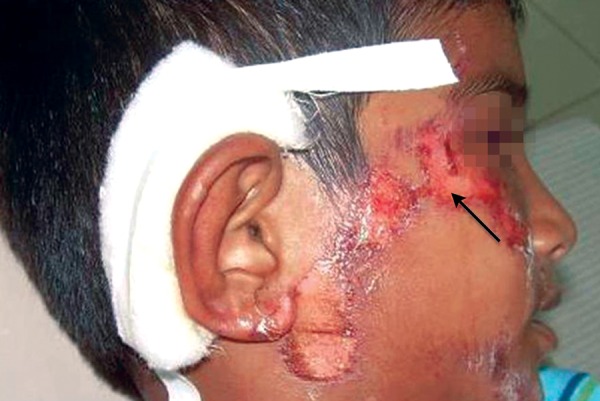
Negligence of school authority

**Fig. 2:**
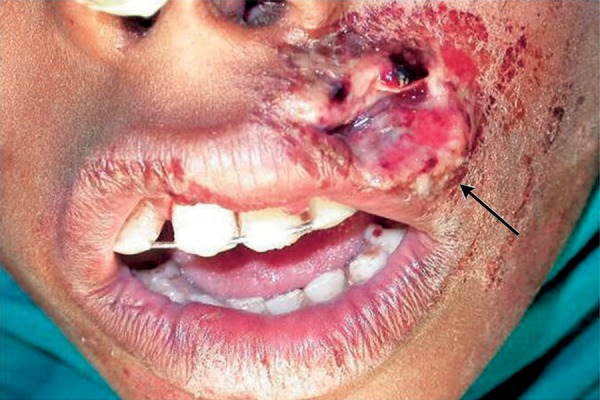
Stone pelting

**Fig. 3:**
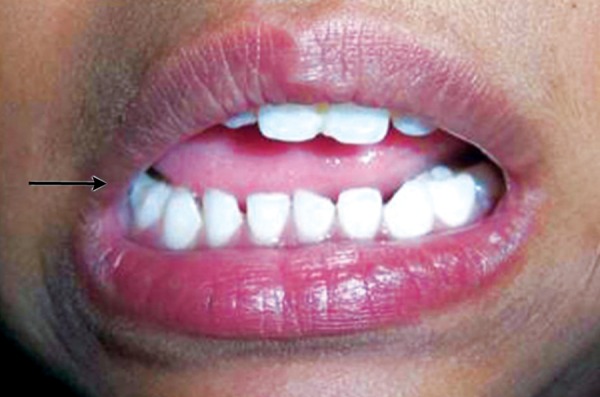
Negligence by the caregiver

**Fig. 4:**
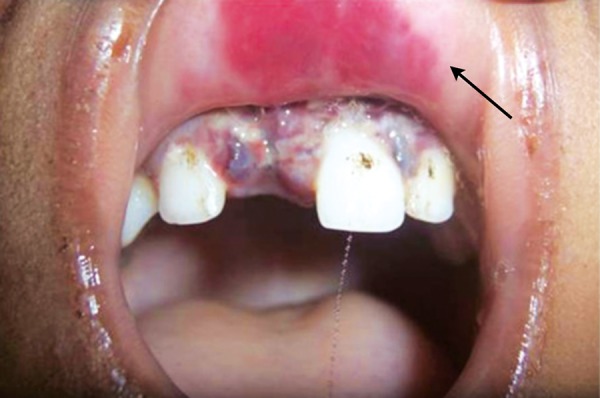
Negligence by the parents

**Fig. 5:**
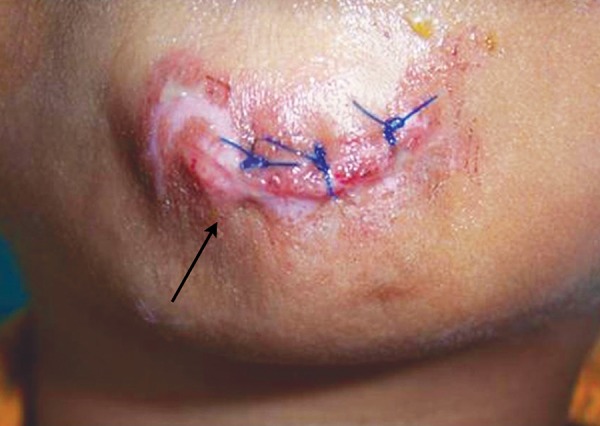
Negligence by local authority

Now focusing on the ignored ones. Negligence on the part of the school authority was clearly depicted in the first case (Fig. 1). As the school playground was not fenced, it was trespassed by private vehicles and the child encountered with an accident. This could have been prevented if the school was well protected. While playing children tend to throw stones or other objects at each other without realizing the imminent consequences (Fig. 2). Parents/ elders’ supervision in general public places or at home helps in reducing these mishappenings. Sometimes doctors themselves are responsible for worsening the situation (Fig. 3). The solution for this is to allow only qualified professionals to practice and regularly audited for the quality of services rendered. Young children tend to fall from bed when put to sleep (Fig. 4). It is the responsibility on the part of the parents to secure their children by having the beds bastioned. Open drainages are a bane to our society. To avoid mishappenings, such half done construction works should be brought to the notice of the representatives of the local authority (Fig. 5).

**Table 1:** **Table 1:** Case reports

*Etiology*		*Age/Sex*		*Clinical presentation*	
*Negligence by*					
1. School authority		10/M		Lacerations on the right middle third region of the face, preauricular and chin region	
		11/M		Soft tissue laceration and dentoalveolar injury	
2. Caregiver		5/F		Ankylosis of the left temperomandibular joint	
3. Parent		2/M		Upper lip hematoma	
4. Local authority		3/M		Laceration on the middle of chin.	
*Child abuse*					
1. Dreadful dreams		7/F		Hematoma over the left middle third region of the face with left periorbital hematoma	
2. Brushing blues		9/F		Broken brush in the left buccal mucosa with laceration	
3. Family conflicts		2/M		Left mandibular parasymphysis fracture.	
*Unpredictable*					
1. Buffalo chase		13/F		Avulsion in relation to both the maxillary central incisors and laceration of the upper lip	
2. Slip of stick		5/F		Laceration in the junction between hard and soft palate	

**Fig. 6:**
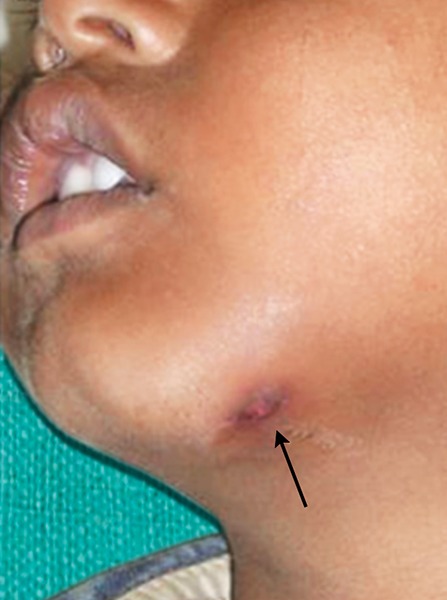
Family conflict

In the present era child abuse has evolved as one of the most evil motives of mankind. The physical and psychological trauma inflicted by the parents or caregivers for every minor, unintentional or undesirable behavior of children is due to their individual internal expectations, inferiority complexes or frustrations. To discipline the children there are certain physical discipline techniques to be followed suggested by Barton^6^ in 1986. Parents/ caregivers should only use hands in corporal punishments. Child should be struck on the buttocks, legs, arms and not on the face. One strike hard enough changes behavior. If the parent strikes more than once it is not to discipline the child but to relieve his/her own frustrations. This is a case of a 2-year-old child who was a victim of a family conflict (Fig. 6).

**Fig. 7:**
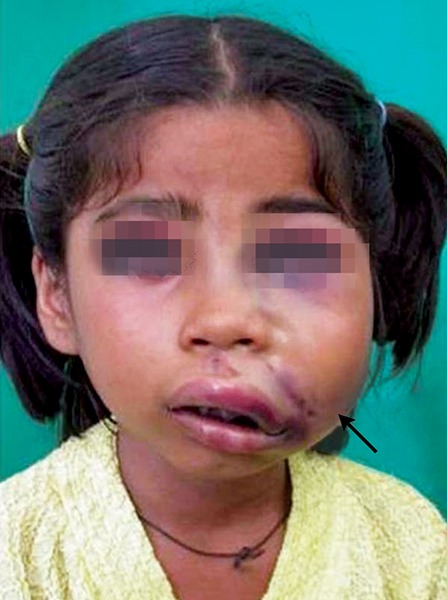
Dreadful dreams

**Fig. 8:**
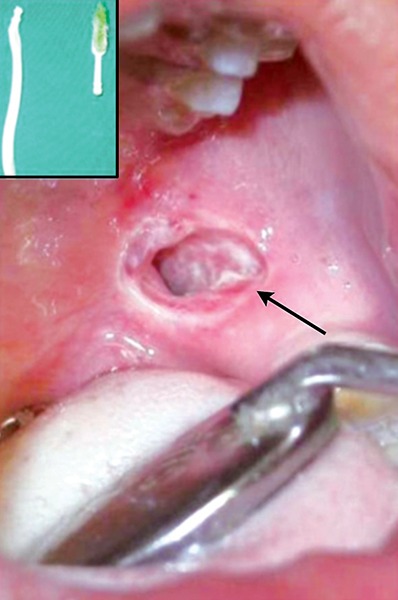
Toothbrush injury

Due to ignorance and superstitious beliefs of the parents, children have to undergo sufferings. Here is an unique case where the father had dreamt that his daughter was the reason for his ill fate and had hit the child when the child was still asleep (Fig. 7). Strict government rules have to be legislated against such kinds of abuse against children. This is another case of child abuse reported wherein the child was beaten up by his father while brushing his teeth resulting in soft tissue laceration of buccal mucosa. The severity can be evidenced by the fact that the toothbrush came out in two pieces (Fig. 8).

**Fig. 9:**
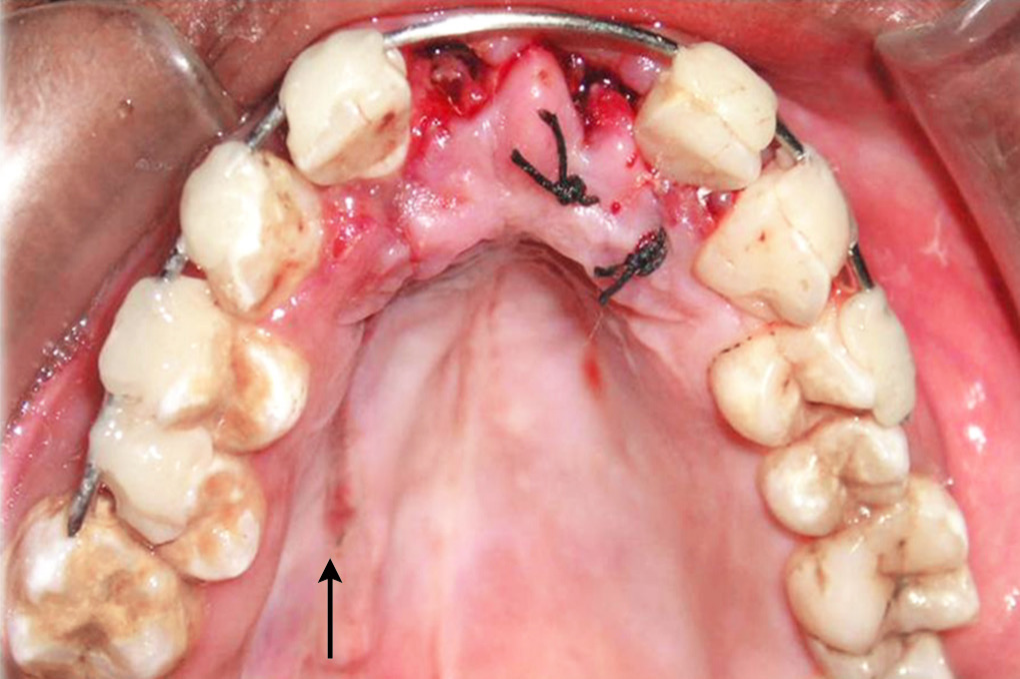
Buffalo hit injury

**Fig. 10:**
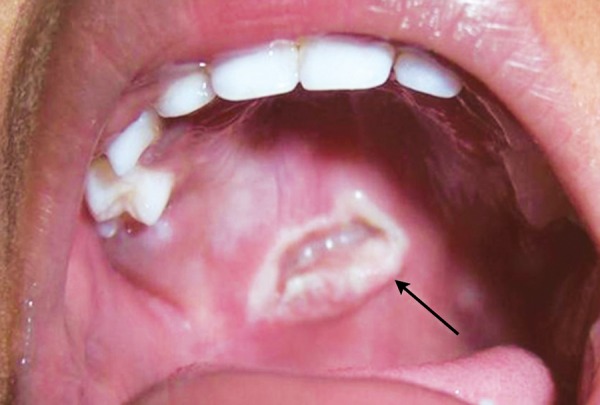
Accidental slip of the stick injuring the palate

There are certain instances where we can prevent or at least reduce their incidence. But certain etiologies are unpredictable and out of our control. Such cases are seen in the rural settings where a child was chased by a buffalo (Fig. 9). Another case reported wherein the child was playing with a stick which accidentally injured the palate (Fig. 10).

## CONCLUSION

Uri Bronfenbrenner said “The tragedy is not what we don’t know. It’s how we ignore what we do know.”

All these cases reported with different etiologies emphasize the importance of disclosing the risk factors and causes of orofacial trauma for effective treatment and prevention. Special emphasis should be given to provide caregivers with relevant education to improve their knowledge and ability to deal with emergency management and prevention of dental trauma. Furthermore, the etiologies of these injuries should be understood.
